# Surface Roughness Characterisation and Analysis of the Electron Beam Melting (EBM) Process

**DOI:** 10.3390/ma12132211

**Published:** 2019-07-09

**Authors:** Manuela Galati, Paolo Minetola, Giovanni Rizza

**Affiliations:** Department of Management and Production Engineering (DIGEP), Integrated Additive Manufacturing Center (IAM), Politecnico di Torino, Torino 10129, Italy

**Keywords:** Ti6Al4V, roughness, electron beam melting (EBM), surface texture, part orientation, metal additive manufacturing

## Abstract

Electron Beam Melting (EBM) is a metal powder bed fusion (PBF) process in which the heat source is an electron beam. Differently from other metal PBF processes, today, EBM is used for mass production. As-built EBM parts are clearly recognisable by their surface roughness, which is, in some cases, one of the major limitations of the EBM process. The aim of this work is to investigate the effects of the orientation and the slope of the EBM surfaces on the surface roughness. Additionally, the machine repeatability is studied by measuring the roughness of surfaces built at different positions on the start plate. To these aims, a specific artefact was designed. Replicas of the artefact were produced using an Arcam A2X machine and Ti6Al4V powder. Descriptive and inferential statistical methods were applied to investigate whether the surface morphology was affected by process factors. The results show significant differences between the upward and downward surfaces. The upward surfaces appear less rough than the downward ones, for which a lower standard deviation was obtained in the results. The roughness of the upward surfaces is linearly influenced by the sloping angle, while the heat distribution on the cross-section was found to be a key factor in explaining the roughness of the downward surfaces.

## 1. Introduction

Electron Beam Melting (EBM) is an additive manufacturing (AM) technique in which an electron beam is used to melt metallic powders [1,2,3]. The process is classified within the powder bed fusion (PBF) category. EBM has shown the potential to work with many material classes such as stainless steel (17-4), tool steel (H13), Ni-based superalloys (625 and 718), Co-based superalloys (Stellite 21), low-expansion alloys (Invar), hard metals (NiWC), intermetallic compounds, aluminum, copper, beryllium, and niobium [1]. Nowadays, titanium and its alloys [1,4,5,6,7,8] represent key materials for several applications in the aerospace and medical fields, wherein EBM machines are used for mass production [3]. Galati and Iuliano [3] provided a comprehensive overview of the mechanisms during the EBM process and the motivation for the adoption of EBM in mass production. The EBM process consists of different steps. After the powder distribution and before the melting phase, two subsequent steps preheat the powder bed. Murr et al. [9] accurately described the first step, called “preheating one” in the Arcam system, wherein the powder bed is uniformly preheated by a series of beam passages. Then, a further preheating step takes place, in which the preheating area is limited to an offset area of the melting surface. Galati and Iuliano [3] highlighted that while the former preheating aims mainly to avoid the spreading of the powder during the melting phase, the latter decreases the thermal gradient for the subsequent melting phase. Mahle [10] pointed out that the following step is a balancing phase also known as “postheating”. Depending on the total amount of heat supplied during the previous steps, the layer could be cooled down or further heated with the same strategy as the second preheating step. For simulation purposes, Cheng et al. [11] analysed the effects of the preheating phase, which sinters the powder particles and improves the heat transmission between the melted line and the surrounding area during the melting phase. Weiwei et al. [12] observed that the sintered powders also have a certain level of strength, and thus the number of surfaces that need to be supported during the building is reduced. The whole process occurs under a vacuum. The preheating phase and the vacuum environment assure high temperatures and low thermal gradients during the process. For this reason, in their comparison of AM processes for Ti alloys, Froes and Dutta [13] indicated that the EBM parts required no stress-relieving operation. However, Sigl et al. [14] underlined that the improved heat transfer facilitates the adhesion of unmelted powder particles to the component surface.

In their recent work, Leach et al. [15] provided a comprehensive and wide review of metrology in the field of AM processes for metal components. The authors reviewed the current technologies, standards, and benchmarking activities to evaluate the machine accuracy in terms of geometrical and dimensional tolerances and roughness. The authors highlighted the needs, the importance, and the difficulties of evaluating the surface characteristics of AM parts, for which the complexity of the generated surfaces represents a challenging aspect. To account for the design complexity of the surfaces produced through AM techniques, Brown et al. [16] suggested the use of a multiscale approach in which different characterisation parameters are considered to describe and evaluate the surface characteristics. Townsend et al. [17] further reported the techniques that are available for evaluating the surface characteristics of AM components. Both contact and non-contact techniques are employed for the measurement of the surface roughness, but the most widely adopted instrument remains the profilometer [17]. Among the non-contact approaches, 3D scanners often require the application of an anti-reflection layer with a variable thickness, depending on the material and the surface texture. The thickness of the spray coating introduces an error in the scan data that is incompatible with roughness measurements [18]. Moreover, the quality and thickness of each anti-reflective layer was highly dependent on the experience of the operator applying the coating [19]. Senin et al. [20] reported X-ray Computer Tomography (XCT) as one promising technology to evaluate areal characteristics. However, in this case, the large reconstruction errors could affect the areal topography measurements, reducing the accuracy of the measurements significantly [15]. Additionally, when compared to the use of profilometers, XCT analysis is less accessible and more expensive in terms of computational time and costs [15].

Townsend et al. [17] also reviewed the literature about the roughness of EBM parts and concluded that the experimental results typically range from 20 to 50 µm. Deligianni et al. [21] showed that high roughness values are extremely advantageous for biomedical implants, and Ponader et al. [22] demonstrated that deeper valleys of the roughness profile promote a better attachment and proliferation of the cells. Another aspect that could promote osteointegration and nutrient flow is the adoption of porous structures. These structures, if well designed, could reduce the recovery time of the patient, and, at the same time, ensure good mechanical properties of the implant [23]. On the contrary, Baudana et al. [2] stated that poor surface quality is undesirable for other applications such as those of the aerospace sector. In fact, sintered and unmelted powders attached to the external surface could be a possible factor in crack initiation [23,24].

Different conditions occur during the production of vertical or horizontal surfaces, which could result in varied surface characteristics. Liu and Shin [25] investigated the as-built EBM parts for which differences between the vertical and top surfaces in terms of morphology and surface texture have been noticed. Murr et al. [26] characterised the surfaces at the top and depicted the typical multiple grooves due to the scanning mode of the melting beam. Koike et al. [27] analysed the vertical surfaces, showing that they are mainly characterised by an exterior rippled appearance with evident stacked particles. Neira Arce [28] investigated the effect of the particle size on the surface roughness and found that the bigger the powder particles, the higher the surface roughness. Karlsson et al. [29] have shown that the use of finer powders increases the possibility of sintered particles stacked on the part.

The process parameters also affect the final roughness of the surface. Safdar et al. [30] detected that the surface roughness decreases for high values of focus offset and scan speed. Moreover, because of a heat accumulation in the part, the surface roughness is higher when the build height and beam current are increased. Jamshidinia and Kovacevic [31] studied the influence of heat accumulation experimentally, and demonstrated that the surface roughness is inversely proportional to the distance among parts. Galati and Iuliano [3] stated that contours can be used in the EBM process to improve the surface roughness, and Galati et al. [32] provided a detailed description of MultiBeam^TM^, showing the difference in the line profile between a continuous line and a line obtained by using the MultiBeam^TM^ strategy. Klingvall Ek et al. [33] provided a thorough investigation of the effects of the scanning strategy, considering different distances between two adjacent contours, the number of contours, and the line energy. Klingvall Ek et al. [33] obtained no influences on the roughness values. Furthermore, Wang et al. [34] investigated the effects of producing a contour by using MultiBeam^TM^ or continuous line strategies in order to improve the surface roughness. However, Wang et al. [34] established that the process window for improving surface roughness is small, and modifying only the processing parameters has no significant effect on the roughness values. To date, all studies have analysed the surface roughness of as-built EBM parts on vertical and horizontal surfaces only [25]. In addition, the EBM surface roughness has been evaluated without any systematic approach.

This work aims to fill the gap of the absence of an investigation on the effects that the surface orientation and the slope have on the surface roughness and morphology of as-built EBM parts. A specific artefact is designed to avoid support structures, and several replicas of Ti6Al4V are fabricated by an Arcam A2X machine (Arcam AB, Mölndal, Sweden)

The surface texture and characteristics are also investigated for the different positions of the single replica on the build plate. Descriptive and inferential statistical methods are applied to investigate whether the surface morphology is affected by process factors, and to develop an analytical model for predicting the surface roughness in EBM.

## 2. Materials and Methods

### 2.1. Design of the Reference Artefact

Townsend et al. [35] proposed three small test artefacts for evaluating the process capabilities of Laser PBF and EBM systems, in terms of resolution and roughness. For the EBM process, the artefacts were also replicated in different positions into the building volume and in two different orientations: Horizontal and vertical. To evaluate the variation of surface texture along the build volume, nine bars were also produced, and the replicas of the artefacts were attached to them. The roughness was evaluated in terms of the arithmetical mean height (Sa) on the horizontal and vertical surfaces of the artefacts. The means of the roughness values were found to be 8.9 and 32.2 µm for the horizontal and vertical surfaces, respectively. However, the EBM process parameters were not reported in this study. Additionally, the influences of the thermal effects caused by the presence of the bars close to the artefacts replicas on the surface roughness have not been discussed [31]. Including surfaces with different slopes, Reeves and Cobb [36], Kim and Oh [37], and Bartkowiak et al. [38] proposed artefacts for evaluating the roughness of polymeric parts produced by AM systems. Except for the building by Selective Laser Sintering systems, the building of the artefact by Reeves and Cobb [36] required the use of support structures, while the artefact proposed by Kim and Oh [37] included only upward surfaces. Both artefacts have a large projected area, and the possibility to replicate the part into the build volume has not been considered. Strano et al. [39] used the artefact proposed by Reeves and Cobb [36] to evaluate the roughness of metal parts produced by Laser PBF systems. The roughness measurements were collected and analysed only for the upward surfaces, while the down-facing surfaces were not evaluated because of the presence of support structures. Udroiu et al. [40] designed a new artefact with several upward and downward surfaces tilted at various angles, which was used to evaluate the roughness for the material jetting process. However, the roughness of some of the downward surfaces was not detected because of the presence of supports. In all of these cases, the presence of the support structures may have had detrimental influence, reducing the aptitude of the part to describe the process capabilities [41].

**Figure 1:**
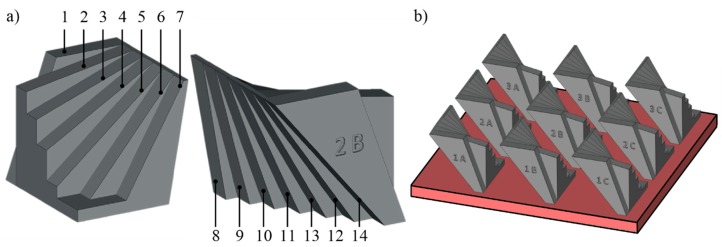
(**a**) Artefact; and (**b**) positions of the replicas on the build plate (single job).

In light of the literature results, the artefact for this study was designed with the aim to evaluate the surface roughness for both downward and upward surfaces. Therefore, the tilted surfaces were designed to avoid support structures. The artefact is thus fan-shaped and includes seven pairs of planes. If the horizontal plane is defined to be parallel to the build plate, the downward-oriented surfaces are tilted with respect to the horizontal plane from 50° to 80° through steps of 5°, while the upward-oriented surfaces are tilted from 0° to 60°, with increments of 10°. Therefore, the angle between each couple of opposite planes decreases from 50° to 20°, with increments of 5°. The surfaces were numbered from 1 to 14, as shown in Figure 1a and Table 1.

The design of the artefact also considered its replication into the same build job to evaluate the process repeatability. In all electron beam-based processes, the quality of the electron beam plays a key role for the process accuracy. For the EBM process, a beam-alignment phase helps to tune the beam quality and must be performed manually by an operator at the beginning of every job. In this phase, the EBM control system divides the build plate ideally into nine equivalent working zones, and the beam alignment is performed on each area. Therefore, the EBM process could perform differently depending on the working area under non-ideal conditions. For the scope of this work, the process repeatability analysis aimed to investigate systematic effects due to the alignment of the beam. As a result, the maximum envelope of the artefact was chosen for fitting at least nine replicas of the artefact into the same build job. Since a square build plate with a side length of 210 mm was used to produce the replicas, the artefact was designed to occupy no more than 50 mm × 50 mm. In this way, each of the replicas of the artefact were also reasonably spaced from each other to avoid heat accumulation.

Figure 1a shows the final design of the artefact, which can be downloaded from Rizza, et al. [42]. The design, as well as the conversion to STL format, was carried out using SolidWorks 2017 software (Dassault Systèmes, Waltham, MA, USA). The STL file contains 94 triangles, and it was generated using a maximum chordal error of 0.0414 mm. For ease of reference, each replica was named by a letter and a number to identify the position of the replica on the build plate. The name was a part of the melted geometry and each replica was positioned with its name toward the machine operator, as can be seen in Figure 1b. The rows were identified by numbers from 1 to 3, while the columns were marked with letters from A to C. The row identified with 1 was the nearest to the door of the machine. The replicas were orientated so that all the surfaces were perpendicular to the rake that spread the powder layer. The build job was prepared using Magics 21.1 (Materialise, Leuven, Belgium). 

### 2.2. Equipment and Material

The samples were produced using an Arcam A2X system with standard Arcam Ti6Al4V powder. The average size of the particles was 75 µm. The layer thickness was fixed to 50 µm. The build job was processed by the EBM build processor 5.0 with a Ti6l4V Standard Theme for the Arcam A2X system. The contour was melted using the MultiBeam^TM^ strategy. The main process parameters are presented in Table 2. The preheating step was set to have a uniform temperature equal to 650 °C. The average current was equal to 12.6 mA.

**Figure 2:**
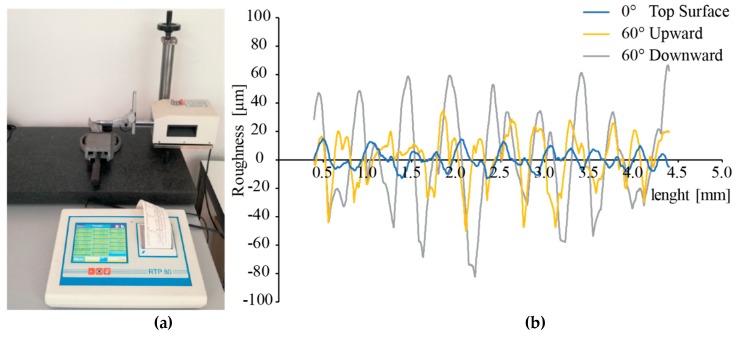
RTP-80 profilometer (Metrology Systems) with a TL90 drive unit and experimental setup for the acquisition of the surface roughness (**a**); and an example of roughness profiles (**b**).

After the production and cleaning of the replicas, surface roughness profiles were measured by a RTP-80 profilometer (Metrology Systems, Volpiano, Italy) equipped with a TL90 drive unit. A cut-off length of 0.8 mm and a sampling length equal to five cut-off lengths (ISO 4288:1997) were used. The experimental setup is shown in Figure 2a. The profiles were acquired in the portion of the surface closest to the top of the replica by positioning the drive unit perpendicularly to the surface. For each surface, three measurements were collected. Since each replica had fourteen surfaces, a total of 378 measurements were carried out, which can be found in the dataset by Rizza et al. [42]. The arithmetic average value (Ra) and root mean square deviation (Rq) were used as descriptors for the surface roughness for the subsequent analyses. Figure 2b shows the typical roughness profiles that were acquired on different surfaces of the EBM specimens.

## 3. Results and Discussion

**Figure 3:**
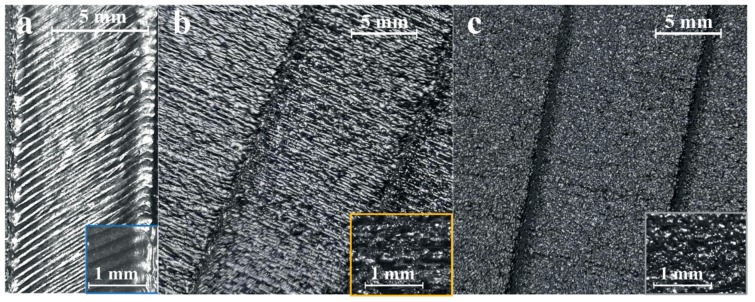
Surface textures observed for the top surface (**a**), upward surfaces (**b**), and for the downward surfaces (**c**).

As can be noticed in Figure 2b, the roughness profile for the upward surface (yellow line) appears to be almost periodic, while the profile for the downward surface (grey line) appears more irregular. This difference may be ascribed to the staircase effect, which is evident for upward surfaces (Figure 3b), while the roughness of downward surfaces appears to be affected by the adhesion of the powder particles (Figure 3c). The profile for the top surface (blue line in Figure 2b) is regular and periodic. It can be noticed that the single-line scans that cause the multiple grooves are visible (Figure 3a). The roughness value (Ra) for the top surface is about 6 µm.

### 3.1. Analysis of the Experimental Validity Using Ra Values

**Figure 4:**
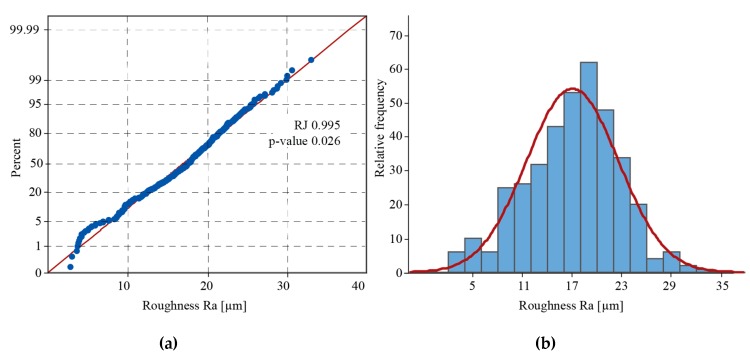
Analysis of the data distribution through (**a**) the Ryan–Joiner test and (**b**) a relative frequency histogram.

The Ryan–Joiner test was performed to verify the normality of the data distribution (Figure 4a). Since the correlation value (RJ) between the collected measurements and the normal scores of the data is approximately equal to 1, the population can be considered normal. The plot of the relative frequency compared with a normal distribution (Figure 4b) shows a slight negative skewness that is typical for full surfaces [43]. The skewness coefficient of the surface texture (Rsk) was equal to −0.24. The negative value maps the process, meaning that the valleys are quite a lot deeper than the heights of the peaks, as established by Horváth, et al. [44].

To prove the effectiveness of the experiments, several statistical hypothesis tests were used. The level of confidence was set to 95%. Two issues were investigated by using analyses of variance (ANOVAs) on the surface roughness, expressed by the Ra value.

The first analysis of variance (ANOVA) was used to determine if there is any systematic effect of the arrangement of the replicas on the build plate on the surface roughness. Therefore, ANOVA evaluated the possibility to consider the replicas of the artefact as replications for the statistical analysis. The inferential statistics were used to determine whether there were differences among the measurements collected by each replica. The statistical query was: “Does the position of the replica on the build plate influence the surface roughness of all the surfaces of the specimens?”. For this analysis, the measurements were grouped into nine groups of 42 measurements each, according to the position of the replica.

The second issue concerns the choice of the sloping angle as a descriptor of the surface roughness. The aim was to investigate whether the chosen sloping angle affects the surface roughness. The statistical query was: “Does the sloping angle affect the surface roughness?”. The measurements were grouped into 14 groups of 27 profiles each, according to the sloping angle.

The last ANOVA inquired about the differences between the downward and upward surfaces. The statistical query was: “Does the direction of the surface affect the surface roughness?”. For these tests, the measurements were grouped from one to seven for the upward surfaces, and from eight to 14 for the downward surfaces, obtaining two groups of 189 profiles each.

The results of the ANOVAs are presented in Table 3.

The ANOVA of the influence of the arrangements of the replicas on the build plate pointed out that there were no relevant differences between the data sets. Therefore, each built part was considered as a replica of a unique artefact. The F-test showed that, with a risk level of 5%, the null hypothesis could not be rejected because the F-critical value (Fmax) was higher than F-ratio. Therefore, no significant differences were noticeable between the data sets. The ANOVA of the influence of the sloping angle on the roughness showed systematic differences among the data sets. Therefore, the chosen sloping angles were significant in explaining the surface roughness. The ANOVA of the influence of the direction (upward and downward) of the surface on the roughness showed systematic differences among the data sets, confirming the experimental observations of the surface textures in Figure 3. Therefore, for the subsequent analyses, the upward surfaces were treated separately from the downward surfaces. As a first result, the downward surfaces appeared rougher and with a lower standard deviation with respect to the upward ones. The higher deviation for the upward surface may indicate a strong effect of the sloping angle on the surface roughness, while the low deviation for the downward surface may show a systematic factor (such as sintered particles) that flattens the measurements. A mean value of Ra equal to 14.922 μm with a standard deviation of 0.657 μm was obtained for the upward surfaces, while the mean of the Ra values of the downward surfaces was equal to 19.133 μm with a standard deviation of 0.306 μm.

### 3.2. Upward Surfaces Analysis

The previous test about the significance of the sloping angle was repeated only for the upward surfaces. The test provided evidence that the sloping angle was still strongly significant in describing the surface roughness for the upward surfaces. To forecast the surface roughness based on the sloping angle, a linear regression was performed using Minitab17.1. The predictive model for the Ra values of the upward surfaces of the as-built EBM Titanium surfaces is given by Equation (1):

(1) Ra [μm]=6.103+0.294×angle

where the variable “angle” is expressed in a decimal degree.

**Figure 5:**
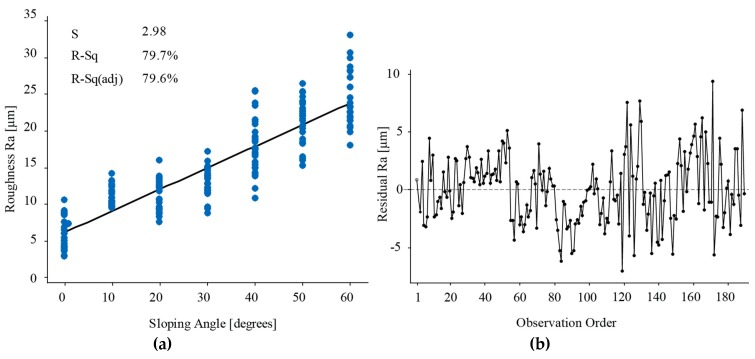
Linear regression model for the roughness of the upward surfaces.

As shown in Figure 5, the R-squared adjusted and R-squared values were high, and therefore the model fits the data set well. Even the residuals were structureless and did not contain any specific pattern. Therefore, the independence assumption on the residuals was fulfilled for the applied method.

The predictive model for the Rq values of the upward surfaces of the as-built EBM Titanium surfaces is given by Equation (2):

(2) Rq [μm]=7.372 + 0.361×angle

where the variable “angle” is expressed in a decimal degree. The R-squared and the R-squared adjusted values are equal to 81.99% and 81.89%, respectively. These high values demonstrated that the regression model fits well the experimental data.

The results confirm that both the linear regression models describe the role that the sloping angle plays in the roughness of the upward surfaces well. The extremely high values for R-squared and R-squared adjusted indicate that the sloping angle represents a good predictor for the variation of the surface roughness.

### 3.3. Downward Surfaces Analysis

The previous test about the significance of the sloping angle was repeated for the measures collected on the downward surfaces using both the Ra and Rq values. The inferential tests did not provide enough evidence for the downward surfaces. Consequently, the sloping angles may not explain the surface roughness, and for this reason it was not possible to define a predictive model. Therefore, the variation of the surface roughness could be due to other process factors.

**Figure 6:**
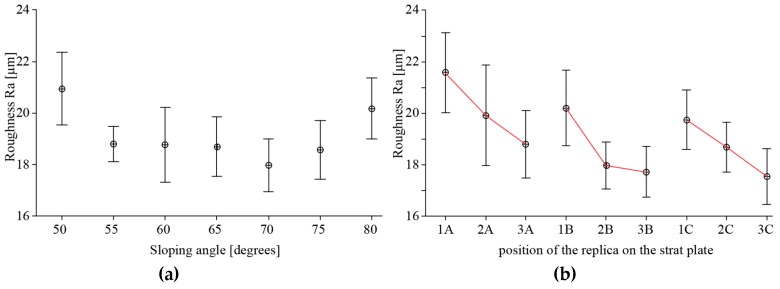
Interval plot for surface roughness for the downward surfaces according to the slope (**a**) and the position on the build plate (**b**).

**Figure 7:**
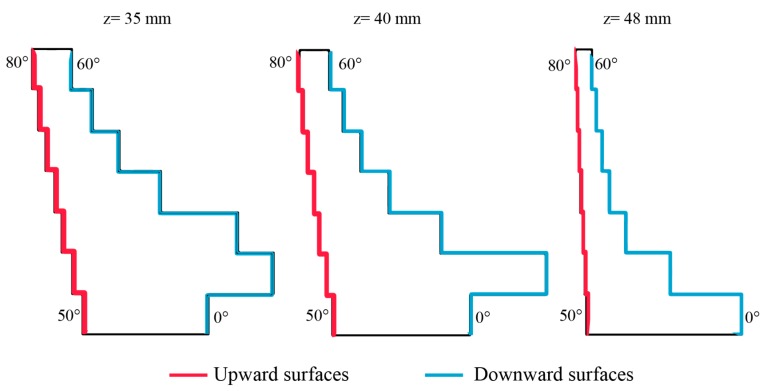
Melting areas at different build heights of the artefact (single replica).

A two-way analysis of variance on Ra values evaluated whether there are effects of the slope and the positions of the replicas on the surface roughness. Both factors and their interaction were significant according to the results of the p-value and F tests (Table 4). However, since the intervals overlap each other (Figure 6), the sloping angle and the positions of the replicas were not significant for the variance of the surface roughness, but they were significant for the mean. In fact, the roughness values were quite similar except for the surfaces tilted at 50° and 80°, for which the difference in the mean appears to be more relevant. This difference could be explained by analysing the heat distribution in the melting area during the EBM process. Figure 7 shows the cross-section of a single replica at different build heights which coincides to the melting area at that height. For the surface tilted at 50°, the melting area for each layer is large and almost constant along the building axis. Instead, the melting area for the surface tilted at 80° is small and decreases rapidly along the building direction. A larger melting area means a higher amount of heat accumulated locally during the process and a lower cooling rate. The dissipation of this heat through the external surface towards the adjacent powder may cause more powder particle adhesion. For the smaller melting areas, the heat accumulated during the melting phase is lower and the cooling rate is higher. For these surfaces, the additional heat supplied during the post-heating phase, to globally balance the amount of heat supplied during the melting and the preheating phases, may significantly increase the local temperature. These heating cycles may cause further sintering of the powder particles closer to the surface of the part and increase the roughness value. Additionally, a certain trend of the mean of the roughness values can be observed by grouping the replicas according to the column. The red lines in Figure 6b highlight a decreasing of the surface roughness when the replicas are nearer to the backside of the machine. The roughest surfaces appear to be those closest to the opening door of the built chamber of the Arcam A2X machine. This result can be explained again by differences in the temperature distribution of the layer and a different cooling rate due to the arrangement of the replicas on the build plate.

## 4. Conclusions

In this work, an innovative artefact was designed to analyse the effects of the sloping angle and the building direction on the surface roughness for as-built EBM parts made of standard Ti6Al4V, using an Arcam A2X machine. The artefact was also designed to be replicated in the same job to investigate the replicability of the process. Consequently, nine replicas of the artefacts were built. The main findings can be summarised as follows:

- No evidence of an effect of the arrangement of the replicas on the build plate was found for the upward surfaces.
- The upward surfaces appeared less rough compared to the downward ones that, on the contrary, showed a lower standard deviation.
- The mean roughness value (Ra) for the top surfaces was found to be around 6 µm. Mean Ra values were found to be around 15 and 19 μm for upward and downward surfaces, respectively. These values are in line with those of the literature on the EBM process, meaning that the artefact is an adequate representative of the process.
- The surface roughness of the upwards surfaces was mainly influenced by the staircase effect, and thus by the slope angle. The surface roughness was linearly dependent on the sloping angle.
- The heat distribution affected the surface roughness for the downward surfaces. The surface roughness appeared to be independent of the sloping angle, due to the differences in the heat distribution during the melting of the section of the designed reference.

The heat distribution on the cross-sectional area is deemed to be a key factor causing the adhesion of unmelted powder to the surface parts. This may explain the results of the previous literature studies, in which no strong relation was found among the process parameters and the surface roughness.

In light of these results, surface orientation should be considered carefully during the part and job designs. The orientation of the surface in the upward direction is always preferable with respect to the downward one. Additionally, for upward surfaces, sloping angles lower than 30° should be preferred. For downward surfaces, the heat transfer that causes the particle adhesion has to be considered. Small variations in melting areas are recommended to avoid non-uniform heat distribution during the EBM process.

Further future investigations aim to design a new artefact in which the melting area is constant for each layer, and each corresponding tilted surface might be used to identify whenever the sloping angle for the downward surfaces and the process parameters influence the surface roughness. A better understanding of the surface roughness could help during the design and process selections for industrial applications and drive the optimisation of the part orientation.

## Figures and Tables

**Table 1:** Identification of the surfaces on the artefact.

**Upward Surfaces**						
1	2	3	4	5	6	7
0°	10°	20°	30°	40°	50°	60°
**Downward Surfaces**						
8	9	10	11	12	13	14
80°	75°	70°	65°	60°	55°	50°

**Table 2:** Process parameters.

**Process Parameter for Contour**						
Melting strategy	Scan speed [mm/s]	Focus Offset [mA]	Beam Current [mA]	Number of spots	Number of contours	Hatch contours [mm]
MultiBeam	850	6	5	70	3	0.29
**Process Parameter for the Hatching**						
Melting strategy	Speed Function	Focus Offset [mA]	Beam Current Max [mA]	Reference Length [mm]	Reference Current [mA]	Line Offset [mm]
Continuous	45	25	20	45	12	0.2

**Table 3:** ANOVAs on the raw data [42]. Analysis 1: ANOVA of the influence of the arrangements of the replicas on the build plate. Analysis 2: ANOVA of the effect of the sloping angle on the surface roughness. Analysis 3: ANOVA of the influence of the direction (upward and downward) of the surface on the roughness.

Analysis	Null Hypothesis: The position of the replica on the build plate affects the roughness	Degrees of Freedom	Variance	F-ratio	Fmax
1	Examined factor	8	6.89 × 10^−3^	0.22	1.96
	Casual errors	369	3.14 × 10^−2^		
	Total	377			
2	Examined factor	13	6.64 × 10^−1^	79.96	1.75
	Casual errors	364	8.31 × 10^−1^		
	Total	377			
3	Examined factor	1	2.08 × 10^−1^	9.98	3.93
	Casual errors	106	2.08 × 10^−2^		
	Total	107			

**Table 4:** Two-way analysis of variance for the downward surfaces.

Source	Degrees of Freedom	Sum of Square	Mean Square	F-ratio	P-value
Slope	6	175	29.2	4.67	0.000
Position	8	300	37.6	6.01	0.000
Interaction	48	520	10.8	1.17	0.008
Error	126	787	6.3		
Total	188	1783			
S	2.5	R-Sq	34.13%		
